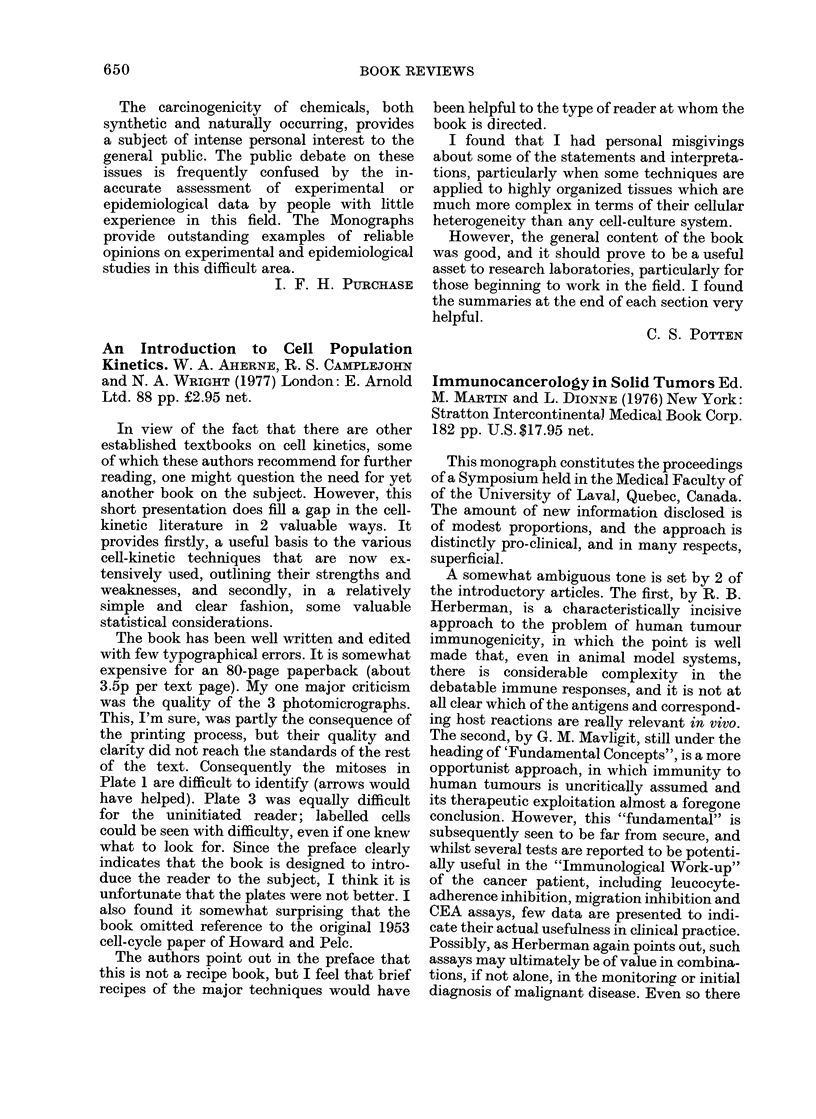# An Introduction to Cell Population Kinetics

**Published:** 1978-04

**Authors:** C. S. Potten


**An Introduction to Cell Population Kinetics.** W. A. AHERNE, R. S. CAMPLEJOHN and N. A. WRIGHT (1977) London: E. Arnold Ltd. 88 pp. £2.95 net.

In view of the fact that there are other established textbooks on cell kinetics, some of which these authors recommend for further reading, one might question the need for yet another book on the subject. However, this short presentation does fill a gap in the cell-kinetic literature in 2 valuable ways. It provides firstly, a useful basis to the various cell-kinetic techniques that are now extensively used, outlining their strengths and weaknesses, and secondly, in a relatively simple and clear fashion, some valuable statistical considerations.

The book has been well written and edited with few typographical errors. It is somewhat expensive for an 80-page paperback (about 3.5p per text page). My one major criticism was the quality of the 3 photomicrographs. This, I'm sure, was partly the consequence of the printing process, but their quality and clarity did not reach the standards of the rest of the text. Consequently the mitoses in Plate 1 are difficult to identify (arrows would have helped). Plate 3 was equally difficult for the uninitiated reader; labelled cells could be seen with difficulty, even if one knew what to look for. Since the preface clearly indicates that the book is designed to introduce the reader to the subject, I think it is unfortunate that the plates were not better. I also found it somewhat surprising that the book omitted reference to the original 1953 cell-cycle paper of Howard and Pelc.

The authors point out in the preface that this is not a recipe book, but I feel that brief recipes of the major techniques would have been helpful to the type of reader at whom the book is directed.

I found that I had personal misgivings about some of the statements and interpretations, particularly when some techniques are applied to highly organized tissues which are much more complex in terms of their cellular heterogeneity than any cell-culture system.

However, the general content of the book was good, and it should prove to be a useful asset to research laboratories, particularly for those beginning to work in the field. I found the summaries at the end of each section very helpful.

C. S. POTTEN